# Youths with disabilities: Utilization and predictors of Youths Friendly Reproductive Health Services in Dessie City Administration, North East Ethiopia, 2021: A cross‐sectional study

**DOI:** 10.1002/hsr2.1386

**Published:** 2023-07-04

**Authors:** Eshetu Y. Shehu, Prem Kumar, Wondwossen Yimam, Samuel Anteneh

**Affiliations:** ^1^ Department of Adult Health (N), College of Medicine & Health Sciences Wollo University Dessie Ethiopia; ^2^ Department of Comprehensive (N) College of Medicine & Health Sciences, Wollo University Dessie Ethiopia

**Keywords:** disabilities, predictors, utilization, Youth Friendly Reproductive Health Services

## Abstract

**Background and Aims:**

Younger generations with disabilities are more likely to be affected and have severe difficulties receiving specific services. Ethiopia is no exception to the global trend of poverty‐stricken countries with a higher frequency of illness or disability. This study aimed to assess the utilization of Youths Friendly Reproductive Health Services (YFRHS) and predictors among youths with disabilities in Dessie City, North East, Ethiopia, 2021.

**Methods:**

A community‐based, cross‐sectional study was conducted. Data were collected from the literature using questionnaires. Bivariable analysis was performed for each independent variable with a *p* < 0.25 on the data imported to multivariate logistic regression analysis. Adjusted odds ratio (AOR) with a 95% confidence interval (95% CI) at a 5% level of significance has measured the strength of association between utilization of youth‐friendly reproductive services among disabilities and independent variables.

**Results:**

Of 423 participants, 91% responded. About 42% of participants had used YFRHS. Youths in the age group of 20–24 years were 2.8 times more likely to use such services than 15–19‐year‐olds (AOR = 2.8, 95% CI: [1.04, 7.44]). Disabled youths living alone were 3.6 times more likely to use the services than those with parents (AOR = 3.6, 95% CI: [1.36, 9.35]). Youths with a visual impairment were 80% less likely to use the services than youths with hearing impairments (AOR = 0.2, 95% CI: [0.18, 0.30]), and disabled youths with poor knowledge were 90% less likely to use the services than participants with good knowledge (AOR = 0.1, 95% CI: [0.01, 0.61]) were statistically significant.

**Conclusion:**

The utilization of YFRHS among youths with disabilities in Dessie Town was low. Participants aged 20–24 years, who lived alone, had visual impairment, and had poor knowledge, were found to be significantly associated.

## 1 INTRODUCTION

Globally, youth with disabilities (YWDs) is estimated to be around 1.3 million, accounting for about 16% of the world's youth population affected by disability, with one in five Ethiopians under the age of 15 years having some form of disability.^1^, ^2^ In Nigeria, a 2021 estimate by the United Nations Population Fund suggested that ~53.5% of people with disabilities were utilizing reproductive health services.^3^ A study conducted in Ghana revealed that about 7 out of every 10 students aged 15–24 years had ever utilized sxual and reproductive health (SRH) services.^4^ The SRH utilization among Arba Minch Secondary and Special Need Schools students with disabilities were 40.52%.^5^ Disabled youths are expected to make use of these services more than the general population, as they are vulnerable to social stigma, unwanted pregnancy, unsafe abortion, lack of cognizance, poverty, depressive symptoms, poor self‐image, poor esteem, and illiteracy. These barriers can have negative consequences for their physical, psychological, and social well‐being, as well as for their reproductive rights and choices.^6^ In Ethiopia, YWD is estimated to be around 2.4–4.8 million, representing about 3% of the youth population. However, Ethiopia also has low levels of reproductive health knowledge and awareness among the youth, especially among YWD and has limited availability and accessibility of Youths Friendly Reproductive Health Service (YFRHS) for YWD.^7^ There is also a lack of research and data on the utilization and predictors of YFRHS among YWD in Ethiopia.

This study provides evidence‐based information on the utilization of YFRHS among YWDs in Dessie City, Ethiopia, which can inform policymakers, stakeholders, and researchers on how to improve the quality and inclusiveness of these services. As a limitation, in Ethiopia, the majority of previous studies were conducted at the institutional level and among youths in the general population, whereas this study was conducted at the community level, and the study participants were exclusively youths with disabilities. In addition, there are no similar studies in Ethiopia or globally that assess the utilization of YFRHS among disabled youths. This is insufficient to completely recognize and explore utilization and predictors of youth‐friendly reproductive services among youths with disabilities.^8^, ^9^, ^10^, ^11^, ^12^, ^13^, ^14^, ^15^

The clinical statement of the study's findings is that youth access to reproductive health services is essential for addressing the SRH needs of young people with disabilities, who are more vulnerable to a variety of barriers and disadvantages when trying to get high‐quality care. In the study, factors such as youths in the age range of 20–24 years, disabled youths who live alone, disabled youths who have a visual impairment, and disabled youths with poor knowledge are identified as significant predictors. The study can guide policymakers, scholars, and stakeholders on how to improve the SRH interests of physically challenged youths and increase the accessibility and quality of YFRHS.

## 2 METHODS AND MATERIALS

### 2.1 Study area design and period

The research was conducted in Dessie, which is located 401 km from Addis Ababa. In 2016, the entire population was 311,724 people, according to the city administration, and 87,904 of those 87,904 are youngsters aged 15–24 years old. The city has six hospitals, two of which are government and four of which are private; eight health centers with YFRHS; and two nongovernmental health institutions. According to the Dessie municipal administrative report, the city has 817 youths with disabilities, among whom 250 have impaired mobility, 242 have hearing and language impairments, 241 have visual impairments, and 84 have multiple impairments. A community‐based cross‐sectional study was conducted from March 2021 to June 2021.

### 2.2 Source and study of population

All disabled youths who were living in Dessie City Administration were the source population, and all randomly selected disabled youths who were living in Dessie City Administration were the study population.

### 2.3 Inclusion and exclusion criteria

All disabled youths aged 15–24 years who have resided in the Dessie City Administration for at least 6 months, and those who were not present at the time of data collection and were critically ill during the study period, were excluded.

### 2.4 Sample size determination

The sample size was determined using single population proportion formula:

$$n=\frac{z^{2}(1-\alpha /2)p(1-p)}{d^{2}}\frac{{\;z}^{2}\left(1-\alpha /2\right)p(1-p)}{d^{2}}=\;\frac{{\left(1.96\right)}^{2}0.5\left(1-0.5\right)}{0.05^{2}}=384+10\%=423,$$

where, *n* = sample size, *p* = estimated proportion (50%), *d* = margin of error = (5%), and *α* = level of confidence = 95% (*Z α*/2 = 1.96). By considering a 10% nonresponse rate, the calculated sample size was 423.

### 2.5 Sampling technique and procedure

The list of the number of disabled youths and their corresponding house numbers/phone numbers were obtained from the Dessie City Administrative Office of the Disability Association, and 817 were registered in the 6 clubs with different disabilities, namely impaired mobility, visual impairment, hearing impairment, and multiple disabilities. After obtaining the sampling frame, the first participant was identified using the lottery method and then the systematic sampling technique was used using every two *K* ^th^ intervals (*K*/*n* = 817/403 = 2). Youths who were available and provided verbal consent over the telephone were interviewed.

### 2.6 Dependent variable

Utilization of YFRHS (Yes/No).

### 2.7 Independent variables

Sociodemographic variables: Age, sex, educational status, marital status, religion, ethnicity, and occupation.

Types of disability: Hearing, visual, mobility, multiple impairment, and intellectual dwarfism.

Healthcare‐related factors: Availability of YFRH facility, convenient time, accessibility, and service provider handling.

### 2.8 Operational definitions

Youths with disabilities: In this study, it refers to individuals aged between 15 and 24 years with disabilities, such as long‐term mobility, hearing, and visual impairments, who are living in Dessie City Administration.

YFRHS utilization: It refers to the use of one or more components of basic reproductive health services such as contraceptive use, sexually transmitted infections treatment and diagnosis, pregnancy testing, antenatal and postnatal care, abortion care services, and education and counseling on reproductive health issues within the previous 12 months.

Knowledge: Participants who scored 80%–100% on knowledge‐related questions were considered as having good knowledge, 50%–79% as having moderate knowledge, and poor knowledge with a score of <50% of the correct responses.

Attitude: Study participants who scored greater or equal to the mean score were considered as having favorable attitudes.

Hearing impairment: In this, it refers to the youths with disabilities, that is, partial or full loss of ability to hear in one or both of the ears.

Visual impairment: In this, it refers to the youths with disabilities, that is, the functional limitation of visualization in one or both of the eyes.

Mobility impairment: Refers to the inability of youths with a disability to use one or more of his/her extremities, or a lack of strength to walk, grasp, or lift objects, and utilizing a wheelchair, crutches, or a walker to mobilize.

Multiple impairments: It refers to the youths who have more than one kind of disability, which includes long‐term mobility, hearing, and visual impairments.

### 2.9 Data collection methods and procedures

A pretested, structured, face‐to‐face interviewer‐administered questionnaire was used to collect the data. The questionnaires were close‐ended, adapted by reviewing previous literature, and suited to the local situation. The Content validity of the tool was assessed by two experts who have a specialty in the field of reproductive health. The questionnaires were first written in English, then translated into Amharic, and then back into English. The data were collected by five trained data collectors with sign language and supervised by two clinical nurses.

### 2.10 Data quality assurance

A questionnaire guide was created, and data collectors and managers received training. One week before the real data collection period, 5% of the youths in Kombolcha town participated in a pretest. The questionnaires were adjusted based on the results of the pretest. Experts in reproductive health assessed the questionnaire's validity and content. The collected data were reviewed by the primary investigator and supervisors each evening of the data collection day for consistency and completeness.

### 2.11 Statistical analysis

The data obtained from each respondent were entered using Epi‐data version 4.4.2.1 and exported to IBM SPSS statistics version 25 for analysis. The K‐nearest neighbor imputation technique was used to fill in missing values in data sets by finding and comparing the most similar points given a certain number of nearest neighbors. Frequency, mean, and SD was used to describe the study population with relevant variables. Bivariable and multivariable logistic regression models were used to assess the presence of any association between each independent variable and dependent variable with two‐sided tests. The analyses conducted for this cross‐sectional study were pre‐specified. An investigation into the factors influencing the use of reproductive health services among disabled youths was conducted by the distribution of their population. No additional subgroup analyses were attempted and the null hypothesis for each dependent variable assumed that there would be no disparity in the utilization of reproductive health services among disabled youths. An adjusted odds ratio (AOR) was used to know and ascertain any association between the independent and dependent variables, and significance was declared using a 95% confidence interval (95% CI). Those candidate variables in bivariable logistic regression with a *p* < 0.25 were moved to a multivariable logistic regression model for the dependent variables to control potential confounding variables. All the assumptions for binary logistic regression, that is, model goodness‐of‐fit, were checked by the Hosmer and Lemeshow tests (>0.05), and the multicollinearity test was checked by the Variance Inflation Factor, and it was between 5 and 10, and there was no value showing the presence of multicollinearity between independent and outcome variables. The *p* < 0.05 at multivariable analysis was considered statistically significant for this study.

### 2.12 Ethical consideration

Ethical clearance was obtained from the Department of Adult Health Nursing and the College of Medicine and Health Sciences at Wollo University. Formal permission was obtained from the Dessie Town Health Office administration. Written informed consent was obtained from all the study participants. The code number was used to ensure the confidentiality of the participants. This study was carried out under the Helsinki Declaration's ethical principles.

## 3 RESULTS

### 3.1 Socio‐demographic characteristics of the respondents

A total of 423 youths with disabilities were chosen at random and 386 responded, resulting in a response rate of 91%. The mean age of participants was 20.3 (2.3 SD) years and more than half of them, 218 (56%), were between the ages of 20 and 24 years. More than 218 (56%) disabled youths had completed less than a primary level of education and 254 (66%) were students. Concerning marital status, 293 (76%) of them were single. Regarding disabled youths, those who have a visual impairment account for 129 (33%) and most disabled youths (42.7%) became disabled during their childhood after birth (Table 1).

**Table 1:** Socio‐demographic characteristics of youths with disability in Dessie City from March 2021 to June 2021 (*n* = 386).

Variables	Category	Frequency (*n*)	Percentage (%)
Age (years)	15–19	168	44
	20–24	218	56
Sex	Male	196	51
	Female	190	49
Marital status	Single	293	76
	Married	69	18
	Divorced	21	5
	Widowed	3	1
Educational Ssatus	Unable to read and write	35	10
	Able to read and write only	59	15
	Primary	124	32
	Secondary	136	35
	Diploma and above	32	8
Religion	Orthodox	183	48
	Muslim	167	43
	Protestant	24	6
	Catholic	12	3
Occupation	Student	254	66
	Employed	30	8
	Unemployed	57	15
	Merchant	45	11
With whom do you live	Parents	185	48
	Relatives	71	18
	Husband/wife	51	13
	Alone	79	21
Ethnicity	Amhara	336	87
	Afar	21	5.4
	Oromo	14	3.6
	Tigray	15	3.9
Type of disability	Hearing impairment	109	28
	Visual impairment	129	33
	Mobility impairment	118	31
	Partial multiple impairment	30	8
Time of disability	At birth	129	33.4
	Childhood (after birth to 5 years)	165	42.7
	Above 5 years	92	23.8

### 3.2 Youth‐friendly service utilization of YFRHS

**Figure 1:**
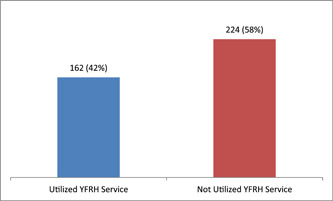
Utilization of Youths Friendly Reproductive Health Services (YFRHS) among youths with disability in Dessie City Administration from March 2021 to June 2021 (*n* = 386).

The study found that 58% of the respondents (95% CI: [49%–64%]) had utilized the YFRHS in the past 12 months (Figure 1).

### 3.3 Reproductive health service utilization and associated predictors

In a bivariable logistic regression analysis, age, occupation, type of disability, living alone, and poor knowledge about youth‐friendly reproductive services were all associated with the use of these services. Those variables that have a value ≤0.25 were entered into a multivariable logistic regression using enter model to adjust for possible confounders.

Participants in the age group of 20–24 years, participants who live alone, have a visual impairment, and have poor knowledge of YFRHS were found to be significantly associated with the use of reproductive health services in a multivariable logistic regression analysis. In multivariable logistic regression analysis, youths in the age group of 20–24 years (AOR = 2.8, 95% CI: [1.04, 7.44], *p* = 0.02) were about three times more likely to utilize youth friendly services as compared with those in the 15–19 years age group.

Disabled youths living alone were about four times more likely than those living with parents to use YFRHS (AOR = 3.6, 95% CI: [1.36, 9.35], *p* = 0.01). Youths with a visual impairment were 80% times less likely to utilize YFRHS than youths with hearing impairments (AOR = 0.2, 95% Cl: [0.18, 0.30], *p* = 0.04). Youths with poor knowledge were 90% times less likely to utilize YFRHS than participants with good knowledge (AOR = 0.1, 95% Cl: [0.01, 0.61], *p* = 0.02) (Table 2).

**Table 2:** YFRHS among disabled youths in Dessie City Administration from March 2021 to June 2021 (*n* = 386).

Variables	Utilized YFS		COR (95% CI)	AOR (95% CI)	*p*
	Yes	No			
Age (years)					
15–19	53 (31.9%)	113 (68%)		1	
20–24	109 (49.5)	111 (50.5)	2.1 (1.36, 2.07)	2.8 (1.04, 7.44)	0.019
Occupation					
Student	99 (39.0%	155 (61%)	0.7	1	
Employed	17 (56.7%)	13 (43.3%)	1.9 (0.95, 4.39)	3.1 (0.90, 10.74)	0.249
Unemployed	19 (33.3)	38 (66.7%)	0.7 (1.23, 4.49)	0.3 (0.09, 1.09)	0.359
Merchant	27 (60.0%)	18 (40.0%)	2.3 (0.43, 1.43)	0.8 (0.23, 2.18)	0.065
With whom do you live?					
Parents	65 (35.1%)	120 (64. %)	0.8	1	
Relatives	28 (39.4%)	43 (60.6%)	1.0 (0.68, 2.11)	0.9 (0.35, 2.37)	0.858
Husband/wife	26 (51%)	25 (49%)	1.6 (1.03, 3.59)	3.0 (0.87, 10.49)	0.082
Alone	43 (54.4%	36 (45.6%)	1.9 (1.29, 3.77)	3.6 (1.36, 9.35)	0.010
Type of disability					
Hearing	43 (41.7%)	60 (58.3%)	0.987	1	
Visual Imp	58 (53.2%)	51 (46.8%)	1.9 (0.92, 2.73)	0.2 (0.18, 0.30)	0.035
Impaired Mob.	46 (39.3%)	71 (60.7%)	0.9 (0.52, 1.55)	0.3 (0.1, 0.89)	0.133
Multiple Imps	15 (26.3)	42 (73.7)	0.4 (0.02, 1.01)	0.5 (0.19, 1.14)	0.804
Level of knowledge					
Poor	101 (39.6)	154 (60.4%)	0.97	0.1 (0.01, 0.61)	0.019
Moderate	33 (50%)	33 (50%)	1.4 (0.05, 14.02)	0.2 (0.05, 0.85)	0.638
Good	28 (42.2%)	37 (57.8%)	1.1 (0.72, 12.42)	1	

Abbreviations: AOR, adjusted odds ratio; CI, confidence interval; COR, crude odds ratio; Imp, impairment; Mob, mobility; YFRHS, Youths Friendly Reproductive Health Services; YFS: Youth Friendly Services. Significant at *p* < 0.05.

## 4 DISCUSSION

This study aimed to assess the utilization of YFRHS and predictors among youths with disabilities in Ethiopia. This study revealed that 42% of disabled youths had used YFRHS.

This implied that the utilization of YFRHS in the study area was poor. Although the study subjects were different, a study conducted in the Gondar zone revealed that the utilization of reproductive health services among reproductive‐age women with disabilities was 33.27%: 27.3% and 8.4% in Sidama state and Jimma, respectively. The difference could be due to the study population. However, to contrast and compare this study's findings, no comparable studies that evaluated the use of YFRHS among disabled youths have been studied globally; hence, findings of this study discussed the universal realities and disabilities with reproductive age women.^16^, ^17^, ^18^

Probable justification could be poor access to reproductive health choices from facility level barrier, social barrier, or provider level barrier. Another possible reason could be a lack of confidence and fear of being seen by friends or family, preferences, sexual activity status, childbearing status, the difference in socioeconomic condition, and lack of comprehensive knowledge and unfavorable attitudes.^19^, ^20^

The results of this study revealed that disabled youths aged 20–24 years were 2.9 times more likely to use YFRHS than individuals between the ages of 15 and 19 years. Consistently, a study conducted in the Gondar zone revealed that the utilization of SRH is more among the age of 18 years and above as compared with ages <15 years. This might be because this is the booming period of reproductive activities and this increases the demand for YFRHS.^16^

In this research finding, disabled youths living alone were 3.6 times more likely to use YFRHS than those living with parents. The difference might be due to no parental pressure, autonomy to take decisions, differences in participants' perception level of stigma, and open discussion skills on SRH issues.^21^

In this study, 53.2% of youths with a visual impairment utilized YFRHS, inconsistently in the Sidama state study 63% of youths with disabilities utilized YFRHS. Utilization of YFRHS among hearing impairment was 41.7% more than the study conducted in the Sidama state (30%). This is probably due to the visually functional impairment to access and make use of it, as they are unable to visit frequently and utilize the youth's SRH services centers.^17^

In this study, 60% of participants with disabilities had poor knowledge and 39.6% utilized the services. These finds are consistent in the of Addis Ababa (66%) participants had a poor knowledge with poor utilization of TFRHS. This could be due to the differences in the level of knowledge or awareness towards youth‐YFRHS.^20^

### 4.1 Implications of the study

This study implies that initiatives targeting disabled youths in Dessie town should focus their efforts on the age group of 20–24 years, those living alone, with a visual impairment, and poor knowledge of YFRHS. Such initiatives could be in the form of providing educational information about YFRHS or creating more accessible locations for providing services. In this way, disabled youths will be more likely to utilize these services.

### 4.2 Limitations

The study was conducted only in Dessie town, so it is challenging to generalize the findings to the general population. It may not show a real cause‐and‐effect relationship and focused on individual perspectives.

### 4.3 Recommendations

Researchers recommend increasing awareness and access among disabled youths and developing outreach programs tailored to the needs of individuals who have visual impairments and poor knowledge of YFRHS. Further comparative studies can be conducted to address the limitations and other research gaps.

### 4.4 Conclusion

The utilization of YFRHS among disabled youths in Dessie town was low. Participants in the age group of 20–24 years, participants who live alone, have a visual impairment, and have poor knowledge of YFRHS were found to be significantly associated with the use of YFRHS.

## AUTHOR CONTRIBUTIONS

**Eshetu Y. Shehu**: Conceptualization; data curation; formal analysis; funding acquisition; investigation; methodology; project administration; resources; software; supervision; validation; visualization. **Prem Kumar**: Conceptualization; formal analysis; investigation; methodology; project administration; resources; software; supervision; validation; visualization; writing—original draft; writing—review & editing. **Wondwossen Yimam**: Conceptualization; formal analysis; methodology; resources; validation; writing—original draft & review. **Samuel Anteneh**: Conceptualization; funding acquisition; project administration; resources; supervision; visualization; writing—original draft & review.

## CONFLICT OF INTEREST STATEMENT

The authors declare no conflict of interest.

## TRANSPARENCY STATEMENT

The lead author Prem Kumar affirms that this manuscript is an honest, accurate, and transparent account of the study being reported; that no important aspects of the study have been omitted; and that any discrepancies from the study as planned (and, if relevant, registered) have been explained.

## Data Availability

The data that support the findings of this study are available on request from the corresponding author's Email greenwater3020@gmail.com. The data are not publicly available due to privacy/ethical restrictions. It contains information that could compromise the privacy of research participants.
